# Knockdown of Simulated-Solar-Radiation-Sensitive miR-205-5p Does Not Induce Progression of Cutaneous Squamous Cell Carcinoma In Vitro

**DOI:** 10.3390/ijms242216428

**Published:** 2023-11-17

**Authors:** Marc Bender, I-Peng Chen, Stefan Henning, Sarah Degenhardt, Mouna Mhamdi-Ghodbani, Christin Starzonek, Beate Volkmer, Rüdiger Greinert

**Affiliations:** Skin Cancer Center, Division of Molecular Cell Biology, Elbe Kliniken Stade-Buxtehude, 21614 Buxtehude, Germany; marc.bender@elbekliniken.de (M.B.); ipeng.chen@elbekliniken.de (I.-P.C.); stefan.henning@elbekliniken.de (S.H.); mouna.mhamdi-ghodbani@elbekliniken.de (M.M.-G.); christin.starzonek@elbekliniken.de (C.S.); beate.volkmer@elbekliniken.de (B.V.)

**Keywords:** skin cancer, cutaneous squamous cell carcinoma (cSCC), cSCC cell lines, simulated solar radiation (SSR), miRNAs, metastasis

## Abstract

Solar radiation is the main risk factor for cSCC development, yet it is unclear whether the progression of cSCC is promoted by solar radiation in the same way as initial tumorigenesis. Additionally, the role of miRNAs, which exert crucial functions in various tumors, needs to be further elucidated in the context of cSCC progression and connection to solar radiation. Thus, we chronically irradiated five cSCC cell lines (Met-1, Met-4, SCC-12, SCC-13, SCL-II) with a custom-built irradiation device mimicking the solar spectrum (UVB, UVA, visible light (VIS), and near-infrared (IRA)). Subsequently, miRNA expression of 51 cancer-associated miRNAs was scrutinized using a flow cytometric multiplex quantification assay (FirePlex^®^, Abcam). In total, nine miRNAs were differentially expressed in cell-type-specific as well as universal manners. miR-205-5p was the only miRNA downregulated after SSR-irradiation in agreement with previously gathered data in tissue samples. However, inhibition of miR-205-5p with an antagomir did not affect cell cycle, cell growth, apoptosis, or migration in vitro despite transient upregulation of oncogenic target genes after miR-205-5p knockdown. These results render miR-205-5p an unlikely intracellular effector in cSCC progression. Thus, effects on intercellular communication in cSCC or the simultaneous examination of complementary miRNA sets should be investigated.

## 1. Introduction

Cutaneous squamous cell carcinoma (cSCC) arises from the degeneration of epidermal keratinocytes and is the second-most common skin tumor after basal cell carcinoma (BCC), with an estimated (not yet captured in most cancer registries) annual incidence of 200,000 to 1,000,000 cases in the United States alone [1,2]. The incidence has increased by 50–200% in the last three decades, and further annual increases are projected for the future, particularly considering demographic changes [3,4]. Metastases or local recurrences develop in few (<5%) patients, but they are often fatal due to the lack of effective therapies [3,5]. The 10-year survival rates for patients with locoregional lymph node metastases and distant metastases have been reported as only 20% and 10%, respectively, in 2019 [5]. Although melanoma is characterized by significantly higher mortality, cSCC contributes to 20% of all skin-cancer-related deaths due to its high incidence [6]. Initial studies with immunotherapies hold promise for improving prognosis [5,7], but a comprehensive understanding of tumor development and progression is crucial to improving patient care and expanding treatment options.

Risk factors for the development of cSCCs include cumulative UV exposure, age, fair skin, immunosuppression, and previous skin cancer conditions [8]. For example, the development of cSCCs in immunosuppressed patients after organ transplantation is increased by a factor of 65 to 250 [2,3]. However, cumulative UV exposure is considered the main risk factor for the general population and is responsible for approximately 93% of all skin cancer cases [9]. This is evident through the higher occurrence of cSCCs in sun-exposed areas, such as the facial region (55%) or the extremities (18%) [10]. Additionally, cSCCs exhibit a high tumor mutational burden (TMB), particularly with UV signature mutations, which leads to loss of function in tumor suppressor genes or gain of function in proto-oncogenes [11,12]. Epigenetic changes in methylation patterns [13] or alterations in miRNA expression in cutaneous SCCs [14] are important additional effectors in cSCC etiology, alongside genetic factors. Understanding the changes in miRNA expression during cSCC development and progression is of particular interest, as miRNAs can be used as biomarkers (liquid biopsies) in body fluids to monitor pathological processes or therapies longitudinally.

miRNAs are conserved, small, single-stranded RNA molecules with a length of approximately 19–24 nucleotides. They originate from non-coding gene sequences and largely regulate the transcription and translation of target mRNAs by binding to the 3′ untranslated region (UTR) of a variety of human genes. They are involved in processes like cell differentiation and cell proliferation as well as carcinogenesis, tumor progression, and metastasis (reviewed in [15]). Several differentially expressed miRNAs have been investigated in human cSCCs. For example, let-7a, miR-9, miR-21, miR-31, miR-135b, miR-142, miR-186, and miR-365 are upregulated, while miR-20a, miR-34a, miR-124, miR-125b, miR-148a, miR-181a, miR-193b, miR-199a, miR-203, miR-204, miR-214, and miR-483-3p have been reported as downregulated [9,16]. miRNAs are sensitive to multiple environmental stimuli (including solar radiation) [17] and in silico analysis proposes differentially expressed miRNA-networks after UVA and/or UVB irradiation to be involved in skin-cancer-associated signaling pathways [18]. Moreover, evidence is emerging that reactive oxygen species (ROS) production by VIS and IRA, as elements of solar radiation, supplements skin damage [19] and aging [20], which are processes related to skin cancer formation.

Thus, we scrutinized miRNA expression in five established cSCC cell lines after chronic SSR exposure (UVB + UVA + VIS + IRA). The function of differentially expressed miRNAs was assessed with network analysis as well as literature research and compared to miRNA expression in cSCC tissue [21]. We found miR-205-5p to be significantly downregulated both after SSR exposure and in cSCC tissue. Therefore, we chose to investigate the miR-205-5p function in cSCC in vitro. However, no phenotypic changes in respect to cell cycle regulation, cell growth, apoptosis, or cell migration could be found, rendering miR-205-5p an unlikely intracellular effector and possibly suggesting intercellular functions in cell communication.

## 2. Results

### 2.1. Differential miRNA Expression of cSCC Cell Lines after SSR-Exposure

We examined the miRNA expression of five cSCC cell lines harboring differences in differentiation status, p53 mutations, tumorigenicity and localization after exposure to SSR. Cells were irradiated with a custom-built irradiation device eight times over a course of four weeks resulting in an accumulated dose of 2 kJ/m^2^ UVB, 100 kJ/m^2^ UVA, 224.24 kJ/m^2^ VIS, and 493.92 kJ/m^2^ IRA. A multiplex approach was used to analyze the expression of 51 miRNAs. miRNAs with fluorescence intensities ≥ 2.5 units (a threshold regarded as reliable expression levels) across cell lines were selected for subsequent analyses (Supplementary Table S1). 

**Figure 1:**
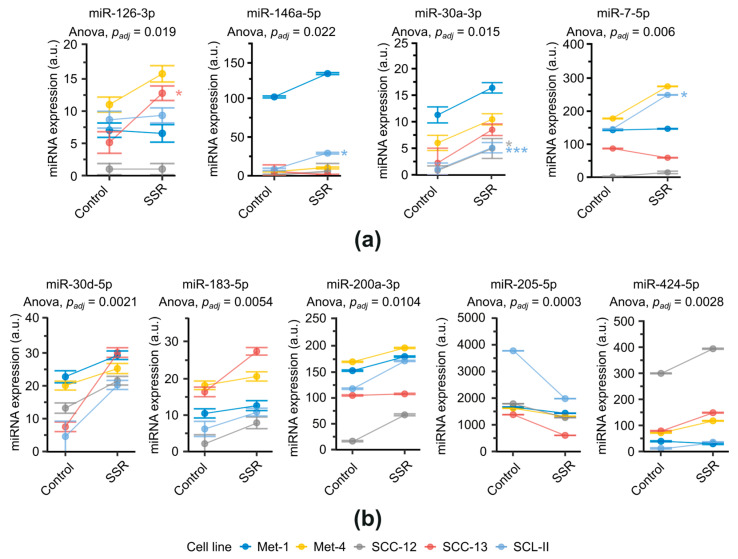
Differential miRNA expression following SSR exposure in cSCC cell lines. miRNA expression measured by flow cytometric quantification (FirePlex^®^-Assay, Abcam). Data are expressed as geometric mean ± geometric SD. *p*_adj_: fdr-adjusted *p*-value. SSR: simulated solar radiation (UVB + UVA + VIS + IRA). a.u.: arbitrary units. *n* = 4. (**a**) Cell-type-specific differential miRNA expression evidenced through a significant interaction term in a two-way ANOVA. Post hoc comparisons were conducted with a Games–Howell test. *: *p* < 0.05; ***: *p* < 0.001. (**b**) Universal differential miRNA expression evidenced by a significant irradiation main effect in a two-way ANOVA with a non-significant interaction term. Due to the absence of significant interaction no post-hoc tests were applied. SSR: simulated solar radiation.

In total, nine miRNAs were differentially expressed post-irradiation (Figure 1) with upregulation of eight miRNAs and downregulation of one miRNA. Two mechanisms of differential expression could be observed, as indicated by two-way ANOVA. The first set of miRNAs showed a significant interaction term, suggesting a cell-type-specific radiation response (Figure 1a). This was accompanied by upregulation of four miRNAs, namely miR-126-3p (fdr-adjusted interaction term of two-way ANOVA, *p*_adj_ = 0.019), miR-146a-5p (*p*_adj_ = 0.022), miR-30a-3p (*p*_adj_ = 0.015), and miR-7-5p (*p*_adj_ = 0.006). A post hoc Games–Howell test showed significant differences for miR-126-3p in SCC-13 (*p* = 0.035), miR-146a-5p in SCL-II (*p* = 0.010), miR-30a-3p in SCC-12 (*p* = 0.020) and SCL-II (*p* = 0.0008), and miR-7-5p in SCL-II (*p* = 0.039). A second set of miRNAs did not manifest a significant interaction. However, a significant irradiation main effect was evident after two-way ANOVA (Figure 1b), suggesting elements of a universal radiation response. In this set, miR-30d-5p (fdr-adjusted irradiation main effect of two-way ANOVA *p*_adj_ = 0.0021), miR-183-5p (*p*_adj_ = 0.0054), miR-200a-3p (*p*_adj_ = 0.0104), and miR-424-5p (*p*_adj_ = 0.0028) were significantly upregulated, whereas miR-205-5p (*p*_adj_ = 0.0003) was significantly downregulated after irradiation. Due to a lack of significant interaction, no post-hoc tests were applied. Results were corroborated by complementary qPCR validation of the results, showing a high concordance of miRNA expression measured via FirePlex^®^ assay and qPCR (Supplementary Figure S1). In summary, in addition to induction of different types of radiation responses, SSR seems promotive rather than suppressive regarding the control of miRNA expression shown by upregulation of 8 out of 9 miRNAs after SSR exposure. 

To obtain an overview of miRNA functions, a network analysis of the induced 8-miRNA set was conducted with miRTargetLink 2.0 (Supplementary Figure S2). This analysis revealed the cooperative regulation of various target genes, including the proto-oncogenes MYC, KRAS, and EGFR (Supplementary Figure S2a). Additionally, the repression of tumor-associated signaling pathways was demonstrated in a gene set enrichment analysis with RBiomiRGS (Supplemental Figure S2b). Among the inhibited pathways, the involvement of the miRNA set in cell growth (mTOR signaling pathway) and various cancer entities (such as prostate carcinoma, melanoma) was noteworthy. 

### 2.2. Investigation of Target Gene Expression after miR-205-5p Knockdown in SCC-12

Bioinformatic network analysis with only one candidate is not sensible. As miR-205-5p was the only downregulated miRNA, we investigated involvement in certain pathways through literature research. This review showed a prominent role of miR-205-5p in various cellular processes associated with carcinogenesis, e.g., regulation of epithelial-mesenchymal transition (EMT), cell proliferation, apoptosis, and cell migration (reviewed in [22]). Additionally, miR-205-5p was the only miRNA concordantly regulated in cSCC tissue samples in a complementary analysis [21]. Therefore, miR-205-5p function in cSCC was scrutinized in knockdown experiments. 

**Figure 2:**
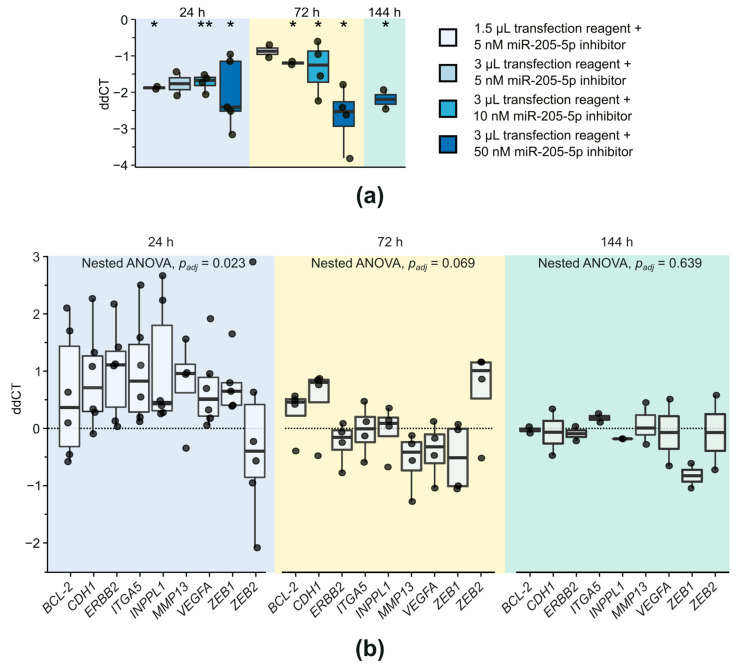
Knockdown of miR-205-5p in SCC-12 leads to transient upregulation of target genes. (**a**) miR-205-5p expression was measured with qPCR at 24, 72, and 144 h post-transfection with a miR-205-5p inhibitor. Data were analyzed with the ddCT method. Results are depicted relative to controls, which were treated with a control sequence. Differential expression was tested using two-tailed one-sample *t*-tests with µ = 0 followed by fdr adjustment. *: *p*_adj_ < 0.05; **: *p*_adj_ < 0.01. *n* = 2–4. (**b**) Target genes of miR-205-5p were measured with qPCR at the same time points and analyzed analogously. Differences in gene expression were tested with a nested ANOVA against µ = 0. A subsequent two-tailed one-sample *t*-test with µ = 0 did not any yield significant results after fdr adjustment. *p_adj_*: adjusted *p*-value. *n* = 2–6.

Knockdown of miR-205-5p was examined by via transfection of SCC-12 cells—showing good transfection capabilities—with different concentrations of a miR-205-5p inhibitor and measurement of miR-205-5p expression via qPCR at different time points (Figure 2a). Transfection led to a significant knockdown of miR-205-5p for the combination of 1.5 µL transfection reagent + 5 nM miR-205-5p inhibitor after 24 h (fdr-adjusted two-tailed one-sample *t*-test with µ = 0, *p*_adj_ = 0.013), 3 µL transfection reagent + 5 nM miR-205-5p inhibitor after 72 h (*p*_adj_ = 0.021), 3 µL transfection reagent + 10 nM miR-205-5p inhibitor after 24 h (*p*_adj_ = 0.003) and 72 h (*p*_adj_ = 0.025), and 3 µL transfection reagent + 50 nM miR-205-5p inhibitor at 24 h (*p_adj_* = 0.013) and 72 h (*p*_adj_ = 0.013) post-transfection. The latter combination showed the strongest reduction in miR-205-5p expression with a 6-fold decrease 72 h after transfection, which was still observable 144 h post-transfection (*p*_adj_ = 0.048), indicating a durable knockdown of miR-205-5p with this transfection approach. Therefore, for subsequent analyses, cells were transfected with 3 µL transfection reagent and 50 nM miR-205-5p inhibitor. 

To test the influence of a miR-205-5p knockdown on target gene expression, qPCR of a set of known targets associated with tumorigenesis (Supplementary Table S3) was conducted (Figure 2b). After 24 h, a significant upregulation of the target gene set could be observed (fdr-adjusted nested ANOVA against µ = 0, *p*_adj_ = 0.023). However, subsequent two-tailed one-sample *t*-tests (µ = 0) did not any show significant differences in single genes after fdr adjustment. Thus, the miR-205-5p knockdown did not lead to strong expression of individual target genes but rather to a moderate upregulation of a target network after 24 h. After 72 h, the expression of target genes began to normalize (*p*_adj_ = 0.069) and dropped back to the baseline level after 144 h (*p*_adj_ = 0.639), indicating that despite prolonged miR-205-5p knockdown, only a transient change in gene expression could be induced. miR-205-5p is presumed to have several hundred target genes [23], and its impact on phenotypic changes cannot be ruled out, despite the rapid restoration of the baseline state for the investigated representative target genes. Therefore, cellular processes associated with carcinogenesis and tumor progression were analyzed following miR-205-5p knockdown.

### 2.3. Examination of Phenotypic Changes after miR-205-5p Knockdown in SCC-12

**Figure 3:**
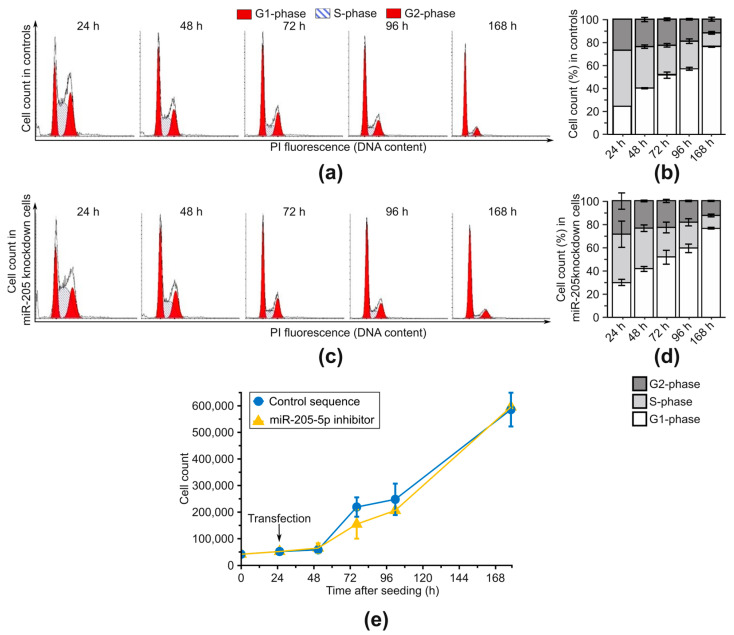
Cell cycle and cell growth remain unchanged after miR-205-5p knockdown in SCC-12. (**a**) Cell cycle distribution of control cells measured by flow cytometry. (**b**) Percentage of cells from (**a**) in each cell cycle phase. (**c**) Cell cycle distribution of cells treated with a miR-205-5p inhibitor measured via flow cytometry. (**d**) Percentage of cells from (**c**) in each cell cycle phase. Significant differences between the cell cycle distribution of treated cells and controls were not evident in a chi^2^ test. Results are shown as mean ± SD. (**e**) Cell growth was measured by counting cell numbers at the indicated time points. The time of transfection is indicated by an arrow. Significant differences could not be detected with a Welch’s *t*-test after fdr adjustment. Results are shown as mean ± SD. *n* = 3.

To investigate the potential influence of miR-205-5p on the proliferation properties of cSCCs, a determination of cell growth and a complementary cell cycle analysis were conducted. SCC-12 cells (representing an aggressive phenotype with p53-deficiency (see §3.1)) were transfected with an miR-205-5p inhibitor and analyzed at various time points. Detached and counted cells were subsequently fixed, and DNA content was measured via propidium iodide staining. After miR-205-5p knockdown, no changes in cell cycle distribution were evident (tested by chi^2^ test on absolute frequencies, Figure 3a–d). Time-dependent changes can be explained by the confluency of cell cultures. The cell growth (Figure 3e) did not differ within the first 48 h, was slightly lower at the time points of 72 h and 96 h miR-205-5p knockdown cells, and then returned to the level of control cells. Testing for differences using Welch’s *t*-test did not lead to detection of significant changes after fdr adjustment. Overall, no evidence was found that miR-205-5p has an influence on the proliferation properties of cSCCs in vitro. 

**Figure 4:**
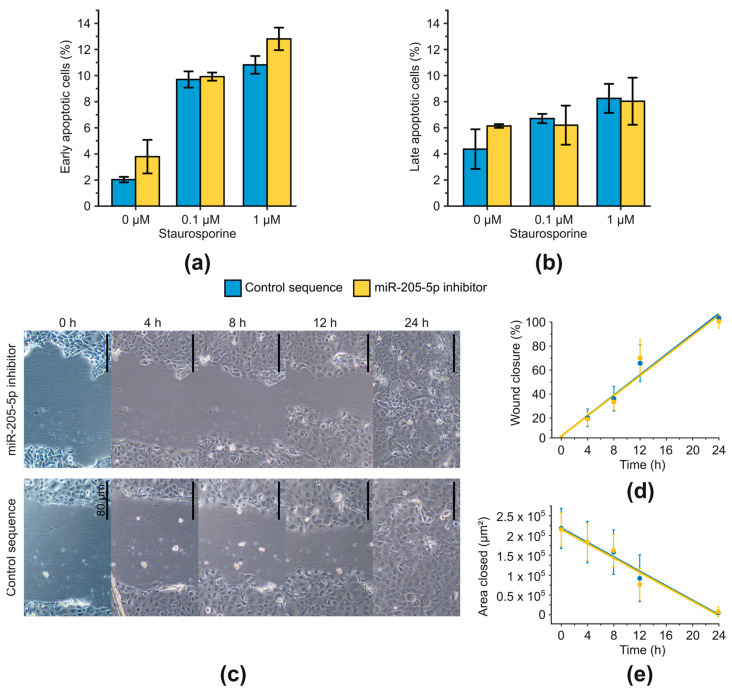
Apoptosis and migration were not affected by miR-205-5p inhibition in SCC-12. (**a**) Early apoptotic cells. Apoptosis was measured flow cytometrically via annexin V assay. Cells were treated with staurosporine for 3 h before quantification of apoptotic cells. (**b**) Late apoptotic/necrotic cells. Apoptosis was measured flow cytometrically via annexin V assay. Cells were treated with staurosporine for 3 h before quantification of apoptotic cells. (**c**) Representative images of a scratch assay. Scratch was induced with a 100 µL pipette tip. Scale bars indicate 80 µm. Wound closure was monitored over a period of 24 h and images were analyzed with the ImageJ plugin wound healing size tool [24]. (**d**) Wound closure in percent. (**e**) Closed scratch area in µm^2^. Significant differences in slopes of linear regression could not be detected with ANOVA for (**d**,**e**). Results are shown as mean ± SD. *n* = 3.

Next, we investigated whether miR-205-5p induces apoptosis or affects apoptosis induction after exposure to a noxious agent (staurosporine). Cells were transfected with a control sequence or miR-205-5p inhibitor and treated with 0.1 μM or 1 μM staurosporine for 3 h. Apoptosis induction was subsequently assessed using the annexin V assay (Figure 4a,b). Repression of miR-205-5p resulted in a slight increase in the proportion of early apoptotic cells (by approximately two percentage points) both in cells without staurosporine treatment and after application of 1 μM staurosporine. The same observation was made for late apoptotic/necrotic cells without staurosporine treatment. However, a chi^2^ test of absolute frequencies showed that none of these changes were significant. In summary, no clear evidence was found for an influence of miR-205-5p expression on apoptosis.

In addition to uncontrolled cell growth and inhibition of apoptosis, cell migration is another essential step in carcinogenesis, particularly regarding tumor progression and metastasis. Therefore, the migratory potential of SCC-12 cells was analyzed after miR-205-5p inhibition using a scratch assay (Figure 4c–e). To ensure that wound closure was solely dependent on migration, cells were incubated with the mitotic inhibitor mitomycin C for 1 h before applying the scratch to inhibit cell proliferation. Images of the wounds were taken at the time points of 0, 4, 8, 12, and 24 h using a bright-field microscope and subsequently analyzed with the ImageJ plugin wound healing size tool [24]. A linear relationship between wound healing or closed area and time was observed, which was not influenced by miR-205-5p inhibition (interaction term of two-way ANOVA, *p* = 0.844). After 24 h, the wounds were completely closed in both conditions. In summary, despite prolonged miR-205-5p knockdown, no phenotypic changes (cell growth, cell cycle, apoptosis, cell migration) were observed in SCC-12 cells.

## 3. Discussion

The precise role of SSR-induced miRNAs in the progression and metastasis of already-transformed cSCC is not well understood. To elucidate the underlying molecular mechanisms, we investigated the expression of miRNAs in five different cSCC cell lines after exposure to chronic SSR. 

Differential expression analysis revealed a cell line-specific upregulation of miR-126-3p, miR-30a-3p, miR-146a-3p, and miR-7-5p after chronic SSR. Similar observations of a cell-specific radiation response (at the mRNA level) were already made between keratinocytes and HeLa cells [25]. Furthermore, a specific radiation response was observed in colon carcinoma (HCT116) and melanoma cell lines (Me45) after UVA irradiation [26]. Recently, our group reported differential expression of several miRNAs in cSCC cell lines in response to different qualities of UV radiation (UVA, UVB and UVA + UVB) [27].

However, the available data on cell-type-dependent differences in cell lines of the same cell type regarding the UV radiation response are insufficient. Nevertheless, this evidence can be supplemented by data from the field of ionizing radiation. In radiation therapy, for example, intratumoral differences in the radiosensitivity of gliomas [28] and esophageal carcinomas [29] have been observed. Additionally, specific radiation responses have been observed in radiosensitive and radioresistant head and neck squamous cell carcinoma cell lines (HNSCC) [30], which share some characteristics with cutaneous SCCs [31]. 

In addition to the specific miRNA alterations, in our investigation, universal upregulation of miR-30d-5p, miR-183-5p, miR-200a-3p, and miR-424-5p, as well as decreased expression of miR-205-5p, could be detected after chronic SSR. Thus, our previous findings of a UV-induced upregulation of miR-183-5p and miR-424-5p were supplemented [27]. Additionally, these observations suggest the involvement of miRNAs in pathways that mediate effects across cell lines as part of a conserved radiation response. In fact, at least parts of the DNA damage response (DDR) are not only comparable within individual tissues or cell types but are conserved between animals, plants, and fungi [32]. Supporting evidence has shown as early as 1994 that the UV radiation response is conserved between mammals and yeast in terms of ras signaling pathway activation [33], and similarities in the UV radiation response have been found between enchytraeids (*Enchytraeus crypticus*) and humans [34]. The similarities in radiation response despite the large phylogenetic distance between the described kingdoms or species underline the importance of a functional (UV) radiation response or DDR, which developed in parts early in evolutionary history. 

In summary, the radiation response in cSCC is composed of conserved elements that encompass similar pathways and proteins across species, as well as elements that are cell-type-specific and related to tumor heterogeneity. Both aspects were reflected in this study by a cell-line-specific or universal miRNA pattern post-irradiation.

To contextualize the described miRNA alterations regarding carcinogenesis, tumor progression, and metastasis, the functions of the differentially expressed miRNAs at the cellular level and in various tumor entities are described in the literature. Due to the cooperative binding to target mRNAs and the associated complex regulation by miRNAs, a network analysis of shared targets has been used to decipher the biological function of miRNAs. A network analysis of the upregulated 8-miRNA set primarily revealed the inhibition of prominent proto-oncogenes such as *MYC*, *KRAS*, and *EGFR* [35] as well as genes involved in the Notch signaling pathway (*NOTCH1*, *NOTCH2*) [36] or cell cycle regulation (*CCNE1*, *CDK6*) [37]. These observations are associated with the inhibition of cell growth signaling pathways (mTOR pathway, WNT pathway), and with tumor suppression in prostate cancer and melanoma in silico, among others. In contrast to this tumor-suppressive profile of the 8-miRNA set, the increased expression of miR-200a-3p in metastatic cSCCs compared to primary tumors [38] and the demonstrated overexpression of miR-424-5p in cSCC through next-generation sequencing (NGS) [39] stand out. This ambivalence suggests a complex miRNA expression pattern in cSCC and a context-dependent function of miRNAs as either tumor suppressors or oncogenes, which has been described for a variety of different cancer entities [40].

Since miR-205-5p was the only miRNA downregulated after irradiation, it was not included in the network analysis. However, its function in special molecular pathways and/or the characterization of target genes of miR-205-5p can be deduced from the literature data. miR-205-5p, together with the entire miR-200 family, is a central regulator of TGF-β-induced epithelial-mesenchymal transition (EMT). Cooperatively, these miRNAs inhibit the transformation to a mesenchymal phenotype by inhibiting ZEB1/ZEB2 expression and thereby preserving E-cadherin expression [41]. On the other hand, the phosphatase SHIP2 (encoded by the *INPPL1* gene) is a target of miR-205-5p. The regulation of SHIP2 by miR-205-5p leads to the activation of the TGF-β/Akt signaling pathway and increased migration rates in keratinocytes [42]. Additionally, miR-205-5p targets a plethora of communication factors, including, e.g., VEGF and FGF1 leading to reduced angiogenesis in gastric cancer [43], whereas exosomal miR-205-5p induces angiogenesis in nasopharyngeal carcinoma by targeting desmocollin-2 [44]. 

Once again, the context-dependent function of this miRNA as a tumor suppressor miRNA or onco-miR becomes evident. For example, it is downregulated in melanoma or prostate cancer but shows increased expression in HNSCC [22]. In cSCC, there is conflicting evidence in the literature. Cañueto et al. describe miR-205-5p as an onco-miR in cSCC, which is correlated with a poorer prognosis for cSCC patients [45]. Consistent with this, a study by Bruegger et al. published in 2013 compared tissue samples from cSCC patients and immunocompetent individuals and found elevated miR-205-5p levels in the tissue of cSCC patients [46]. In contrast, miR-205-5p was expressed at lower levels in metastatic cSCC compared to primary tumors [38]. In a complementary study, we also demonstrated a reduced miR-205-5p expression in cSCC samples compared to skin samples from healthy individuals [21]. Furthermore, re-expression of miR-205-5p in a mouse model inhibited cSCC progression by repressing EMT target genes [47]. On the other hand, Dziunycz et al. did not detect any expression changes in cSCC tissue, but they observed increased miR-205-5p expression after UVA irradiation and a decreased miR-205-5p expression after UVB irradiation in human keratinocytes [48]. 

Overall, miR-205-5p exhibits a complex role in tumors, particularly in cSCC, and is closely associated with the TGF-β pathway and the regulation of EMT. It has also been demonstrated to be inducible (UVA [48]) or repressed (UVB [48], SSR in this study) by UV radiation, highlighting the connection between (UV) radiation and cSCC. Due to the ambivalent role of miR-205-5p and conflicting evidence regarding its function in cSCC, as well as its decreased expression in metastatic cSCC, we decided to further characterize the impact of this miRNA on cSCC progression.

In our investigation, however, the examination of phenotypic changes revealed that miR-205-5p knockdown did not influence cell cycle distribution, cell growth, apoptosis induction, or cell migration. This finding was unexpected, considering that context-dependent activating and inhibiting functions of miR-205-5p on these processes have been described [22]. Nevertheless, the data provide more important information about the role of miR-205-5p in cSCC progression to be further investigated.

miR-205-5p is ubiquitously expressed in the skin [22] and was highly expressed in the five examined cell lines. Knockdown of such highly expressed miRNAs can be challenging [49]. Despite a sustained and up to six-fold reduction in miR-205-5p expression achieved by the miR-205-5p inhibitor used in this study, it cannot be ruled out that the knockdown was insufficient to induce phenotypic changes. However, the effectiveness of this particular miR-205-5p inhibitor is supported by results of De Cola et al., who, using similar transfection conditions and the same miR-205-5p inhibitor, demonstrated both sustained miR-205-5p knockdown and phenotypic changes in breast cancer cells [50]. The absence of any phenotypic changes in our study despite a comparable knockdown can be interpreted in the context of tumor heterogeneity and tissue specific effects. An alternative explanation in context of cSCC progression might be the argument that isolated inhibition of a single miRNA is not sufficient to induce phenotypic changes. This speculation is supported by the results obtained from cSCC tissue [21]. In those samples, in addition to miR-205-5p repression, twelve other miRNAs were downregulated, suggesting that a measurable effect may only occur through the simultaneous modulation of a miRNA set and the cooperative regulation of target genes. Furthermore, it is possible that miR-205-5p plays a role in intercellular communication (e.g., as exosomal cargo) rather than mediating intracellular effects. Tumor cells often utilize this communication to alter the microenvironment in their favor. Prominent examples include the changes in gene expression profiles of fibroblasts to cancer-associated fibroblasts (CAFs) [51] or the modulation of the immune system towards a tolerogenic phenotype [52]. High levels of exosomal miRNAs, including miR-205-5p, and subsequent activation of angiogenesis have been frequently observed in various cancer types [53,54]. While a tumor-suppressive function of exosomal miR-205-5p has been described in breast cancer, the majority of existing evidence suggests the promotion of oncogenic processes by exosomal miR-205-5p. Considering the ambivalent function described in cSCC, the observations regarding the impact of exosomal miR-205-5p on carcinogenesis in other cancer types, and the apparent lack of evidence regarding the role of extracellular miR-205-5p in cSCC, this provides a basis for future research questions.

## 4. Materials and Methods

### 4.1. Cell Culture and SSR Irradiation

Met-1 and Met-4 cell lines (kindly provided by Prof. Boukamp, DKFZ, Heidelberg, Germany) were derived from a primary acantholytic cSCC and a lymph node metastasis, respectively, of the same patient. Met-1 has been shown to be diploid, whereas Met-4 showed hypotetraploid features. Both cell lines are wild type for p53, displayed abnormal differentiation, and showed a similar tumorigenicity of 50% in mice [55]. SCC-12, SCC-13 and SCL-II (kindly provided by Prof. Boukamp, DKFZ, Heidelberg, Germany) descend from primary tumor biopsies of the facial region of different patients. They are p53-deficient in at least one allele, have a higher tumorigenicity in mice than Met-1 and Met-4 (SCC-12/SCC-13: 100%; SCL-II: 64%), and represent tumors of varying differential stages (SCC-13 > SCC-12 > SCL-II) [56,57]. 

Met-1, Met-4, and SCL-II were cultivated in DMEM (Dulbecco’s Modified Eagle’s Medium, Gibco, Paisley, Scotland) supplemented with 10% (*v*/*v*) fetal bovine serum (FBS, #CP18-2361, Capricorn, Erbsdorfergrund, Germany), 100 units/mL of penicillin, and 100 μg/mL of streptomycin (both from Gibco, Paisley, Scotland). SCC-12 and SCC-13 were cultivated in FAD (Mix of DMEM and Ham’s F12 (Sigma-Aldrich, Taufkirchen, Germany) in a ratio of 3:1) supplemented with 5% (*v*/*v*) FBS, 0.05 µg/mL hydrocortisone (Sigma-Aldrich), 5 µg/mL insulin (Sigma-Aldrich, Taufkirchen, Germany), 0.01 µg/mL choleratoxin (Sigmal-Aldrich, Taufkirchen, Germany), and 0.01 µg/mL human epidermal growth factor (h-EGF, Promo Kine, Heidelberg, Germany). 

Cells were cultivated subconfluently and irradiated twice per week with approximately 0.25 MED (minimal erythemal dose) SSR (250 J/m^2^ UVB + 12.50 kJ/m^2^ UVA + 28.03 kJ/m^2^ VIS + 61.74 kJ/m^2^ IRA) over a period of four weeks, resulting in a total dose of 2 kJ/m^2^ UVB, 100 kJ/m^2^ UVA, 224.24 kJ/m^2^ VIS and 493.92 kJ/m^2^ IRA. During irradiation, cells were kept in phosphate buffered saline (PBS, Gibco, Paisley, Scotland) at 35 °C. Controls were treated analogously and incubated in PBS at 35 °C for the irradiation’s duration. 

### 4.2. miRNA Profiling

Seventy-two hours after application of the last irradiation dose, cells were harvested, and miRNAs were isolated with the miRNeasy mini kit (Qiagen, Hilden, Germany) according to the manufacturer’s instructions. Subsequently, miRNA expression was measured via flowcytometric quantification of barcode-labelled miRNA-hydrogel microparticles (Fireplex^®^-Assay, Abcam, London, GB) as previously described [58]. Briefly, five nanograms of purified RNA were added to 35 µL Firefly^®^ particles and incubated for 1 h at 37 °C while shaking (1125 rpm). Particles containing complementary sequences binding up to 60 miRNAs were washed twice with rinse buffer A followed by ligation of universal linkers (60 min, room temperature (RT), 1125 rpm). After washing miR-linker-bound particles with rinse buffers B and A, miR-linkers were eluted with H_2_O at 55 °C and amplified by a linker-specific polymerase chain reaction (PCR). PCR products were transferred back to the particles (60 min, 37 °C, 1125 rpm) and combined with a fluorescent reporter (15 min, RT, 1125 rpm) binding the miR–linker complex. Particle fluorescence corresponding to miRNA expression was measured via flow cytometry (Guava easycyte 8HT, Merck Millipore, Darmstadt, Germany). Raw data were analyzed with the FirePlex Analysis Workbench Software v2.0.274 (Abcam). Expression levels were normalized to the twelve most stably expressed miRNAs. Investigated miRNAs, which were chosen for this study by literature review based on known functions in (skin) cancer, are depicted in Supplementary Table S1. Normalizer miRNAs are shown in bold. 

### 4.3. Quantitative Real-Time Polymerase Chain Reaction (qPCR) for miRNA Detection

qPCR was performed using miRCURY LNA miRNA PCR Assay (Qiagen, Hilden, Germany) according to the manufacturer’s protocol. Briefly, cDNA of the miRNAs (10 ng) was synthesized and analyzed in the qTower^3^ (Analytik Jena, Jena, Germany) with 40 cycles of the suggested PCR program. Amplicon quality was assessed via melt curve analysis. For normalization, the geometric mean of miR-16-5p, SNORD44, and SNORD48 was used. 

### 4.4. qPCR for Gene Expression Analysis

The extraction of total RNA was carried out using the miRNeasy mini kit (Qiagen, Hilden, Germany) in accordance with the manufacturer’s instructions. Subsequently, cDNA synthesis was performed using the SensiFAST^TM^ cDNA Synthesis Kit (Bioline, Luckenwalde, Germany), followed by quantification via qPCR (SYBR Green-containing SensiMix, Bioline, Luckenwalde, Germany). All procedures were performed according to the manufacturer’s protocol. Supplementary Table S2 provides details on the primers used. Expression levels were normalized to the geometric mean of the housekeeping genes β-actin (ACTB), hypoxanthine phosphoribosyltransferase 1 (HPRT1) and TATA-box binding protein (TBP). 

### 4.5. Knockdown of miR-205-5p

Approximately 50,000 cells per well of a 24 well plate were seeded and treated with the HiPerfect Transfection Reagent (Qiagen, Hilden, Germany) one day after seeding. Twenty-five picomol miRCURY LNA miR-205-5p Inhibitor (Qiagen, Hilden, Germany) or 2.5 pmol Negative Control A (Qiagen, Hilden, Germany), 100 µL of FBS-free cell culture medium and 3 µL transfection reagent were mixed, vortexed (10 s), and incubated for 10 min (RT) to form transfection complexes. The transfection mixture was slowly added to each well containing 400 µL fresh medium (with FBS). Twenty-four hours post-transfection, transfection complexes were removed via washing with PBS, and cells were supplied with 500 µL fresh medium and cultivated for subsequent analyses. Knockdown efficiency was checked via qPCR. 

### 4.6. Cell Cycle Analysis

Cell cycle analysis was performed via flow cytometric quantification of propidium iodide (PI, Sigma-Aldrich, Taufkirchen, Germany) fluorescence. After harvesting, cells were transferred to a 15 mL tube, 10 mL cell culture medium was added, and cells were centrifuged for 10 min at 190× g. Cell pellets were resuspended in 10 mL PBS, centrifuged (10 min, 190× *g*) and resuspended in 300 µL ethanol (70%) per 1 × 10^6^ cells. Eighty-five microliters of the cell suspension were transferred to a 96 well plate, mixed with 85 µL tris buffered saline (TBS), centrifuged (3 min, 500× *g*), and resuspended in 160 µL 1× Roti-Block (Carl Roth GmbH, Karlsruhe, Germany) for 5 min. Afterwards, cells were centrifuged again (3 min, 500× *g*), resuspended in 160 µL PI-solution (10 ng/mL), and incubated for 15 min in the dark. Ideally, the DNA content of 10,000 cells was quantified flow cytometrically (Guava easyXyte 8HT, Merck Millipore, Darmstadt, Germany). The analysis was performed with the software guavaSoft v3.1.1 (Merck Millipore, Darmstadt, Germany) and Mod Fit LT^TM^ v6.0 (Verity Software House, Topsham, ME, USA). 

### 4.7. Cell Growth Assessment

Forty thousand cells were seeded into a well of a 24-well plate and either harvested after 24 h or transfected according to Section 4.5. Additionally, cells were harvested at the time points 48, 72, 96, and 168 h post-seeding. Cells were counted using a Neubauer chamber according to standard protocol. 

### 4.8. Annexin V Assay

Cells were transfected according to Section 4.5, cultivated for 24 h and treated with the kinase inhibitor staurosporine in concentrations of 0.1 µM or 1 µM for 3 h to induce apoptosis. Controls did not receive staurosporine treatment. After the 3-h incubation period, the supernatant was collected, cells were harvested and united with the supernatant. The number of cells in the supernatant-cell mixture was counted and cells were centrifuged for 10 min at 200× *g*. The cell pellet was resuspended in 500 µL PBS/10^6^ cells. Then, 125 μL of this solution was transferred to a 96-well plate, centrifuged for 5 min (150× *g*), and resuspended in 100 µL annexin marker solution (VWR, Darmstadt, Germany), followed by incubation for 15 min in the dark. Finally, 80 μL of annexin incubation buffer (VWR, Darmstadt, Germany) was added, and cells were quantified flow cytometrically (Guava easyCyte 8HT, Merck Millipore, Darmstadt, Germany). 

### 4.9. Scratch Assay

The migration potential of transfected cells was assessed via scratch assay 48 h post-transfection to ensure confluency at scratch induction. Cells were transfected according to Section 4.5 and cultivated for another 24 h after removing transfection complexes. After treatment with 10 µg/mL Mitomycin C (Sigma-Aldrich, Taufkirchen, Germany) for 1 h, a scratch wound was applied to the confluent cell layer with a 100 µL pipette tip. Cell debris was removed via washing with PBS. Wound healing was monitored at time points 0, 4, 8, 12, and 24 h after scratch induction under a brightfield microscope (CKX53, Olympus, Hamburg, Germany). Pictures were analyzed with the wound healing size tool plugin [24] in ImageJ v1.53s. 

### 4.10. Statistical Analysis

Statistical analysis was performed in R 4.2.3 or higher. Data were gathered as duplicates, and unless otherwise stated, observations originate from three independent experiments. Results are depicted as mean ± standard deviation (SD), median ± interquartile range (IQR), or geometric mean ± geometric SD. Group comparisons between two groups were performed with a two-tailed Welch’s *t*-test. Categorical data were compared with a chi^2^-test. 

For qPCR analysis, HPRT1 (hypoxanthine phosphoribosyltransferase 1), TBP (TATA box binding protein), and ACTB (actin beta) were used as housekeeping genes for normalization, as previously described [59]. In contrast to Livak and Schmittgen [60], Δ*Ct* and ΔΔ*Ct* values were calculated as:

(1) $${\Delta Ct=Ct}_{ref}-{Ct}_{goi}$$

(2) $$\Delta {\Delta Ct=\Delta Ct}_{ctrl}-{\Delta Ct}_{trt}$$

with *Ct_ref_* representing the *Ct* value of the reference gene, *Ct_goi_* representing the *Ct* value of the gene of interest, Δ*Ct_ctrl_* representing the Δ*Ct* value of the control samples, and Δ*Ct_trt_* representing the Δ*Ct* value of the treated samples. This way, positive/negative Δ*Ct* values directly correspond to higher/lower relative expression, and positive/negative ΔΔ*Ct* values directly correspond to a higher/lower fold change without a need to take inverse values. 

Significance in qPCR analysis was tested with nested analysis of variance (ANOVA) or a two-tailed one-sample *t*-test against µ = 0. Group comparisons for more than two groups were conducted with a one-way ANOVA followed by a Games–Howell post hoc test. For detection of interaction effects between two variables, two-way ANOVA with a subsequent Games–Howell test was utilized. Fluctuations in variances were compensated for by calculating heteroscedasticity corrected covariance matrices in one-way and two-way ANOVA. Results of statistical tests were adjusted for multiple testing using the false discovery rate (fdr) approach developed by Benjamini & Hochberg [61]. Significant results are indicated by asterisks (*: *p* < 0.05; **: *p* < 0.01; ***: *p* < 0.001). 

Gene expression data are shown as log2 values, as they follow a log-normal distribution [62], which enables the use of parametric tests even for small sample sizes. In order to transform values to log2 values, negative gene expression values were set to zero, and one was added to each sample before log-transformation. Gene expression was characterized as differentially regulated if differences were significant and at least 1.5-fold higher than controls. All examined miRNAs represent the human sequence.

### 4.11. Pathway Analysis

Differentially expressed miRNAs were loaded into the web tool miRTargetLink 2.0 [63], and a miRNA-target network was drawn for shared targets. Additionally, the miRNA expression levels as well as corresponding *p* values were used for a pathway analysis with RBiomiRGS [64], enabling the analysis of the influence of the miRNA set on activating or repressing Kyoto Encyclopedia of Genes and Genomes (KEGG) pathways based on a gene set enrichment by logistic regression. 

## 5. Conclusions

In summary, we could show that we can introduce UV-induced changes in a set of miRNAs in cSCC cell lines, with a prominent reduction in expression of miR-205-5p. However, an efficient and durable knockdown of miR-205-5p in cSCC in vitro did not result in changes in cell growth, cell cycle, apoptosis, or cell migration despite the reduced expression of miR-205-5p in cSCC tumor tissue. These observations possibly argue against an intracellular function of miR-205-5p in cSCC and suggest an influence on intercellular processes. Furthermore, the results highlight the necessity of investigating miRNA sets that cooperatively regulate the same target genes rather than analyzing isolated miRNAs.

## Data Availability

The data that support the findings of this study are available from the corresponding author on reasonable request.

## Supplementary Materials

The following supporting information can be downloaded at: https://www.mdpi.com/article/10.3390/ijms242216428/s1. References [65,66,67,68,69] are cited in the supplementary file.
