# Supplementary material for: Knockdown of Simulated-Solar-Radiation-Sensitive miR-205-5p Does Not Induce Progression of Cutaneous Squamous Cell Carcinoma In Vitro
Source: Int J Mol Sci. 2023 Nov 17;24(22):16428. doi: 10.3390/ijms242216428 (PMC10671527; doi:10.3390/ijms242216428)
Supplement: Supplementary file 1 [file ijms-24-16428-s001.zip › Supplementary_tables.pdf]

Supplementary Tables

Table S1. miRNAs examined by FirePlex®-Assay. miRNAs in bold were used for normalization. Asterisks indicate miRNAs with an expression of <2.5 a.u. which were excluded from the analysis.

hsa-let-7c-5p	hsa-mir-26b-5p	hsa-mir-126-3p	**hsa-mir-221-3p**
**hsa-let-7f-5p**	**hsa-mir-29a-3p**	hsa-mir-130a-3p	hsa-mir-296-5p*
hsa-let-7i-5p	hsa-mir-29b-3p	hsa-mir-135b-5p	hsa-mir-378i*
hsa-mir-7-5p	**hsa-mir-29c-3p**	hsa-mir-146a-5p	hsa-mir-424-5p
hsa-mir-9-5p*	hsa-mir-30a-3p	hsa-mir-148a-3p	hsa-mir-495-3p
**hsa-mir-15a-5p**	**hsa-mir-30b-5p**	hsa-mir-155-5p	hsa-mir-503-5p
hsa-mir-19b-3p	hsa-mir-30d-5p	hsa-mir-181a-3p*	**hsa-mir-16-5p**
hsa-mir-21-5p	hsa-mir-31-5p	hsa-mir-181a-5p	hsa-mir-101-3p*
**hsa-mir-22-3p**	hsa-mir-32-5p	hsa-mir-183-5p	hsa-mir-378g*
**hsa-mir-23a-3p**	hsa-mir-34a-5p	hsa-mir-200a-3p	hsa-mir-382-5p*
**hsa-mir-23b-3p**	hsa-mir-34c-5p	hsa-mir-200b-3p	hsa-mir-1246*
**hsa-mir-24-3p**	hsa-mir-92a-3p	hsa-mir-203a-3p	hsa-mir-4454*
**hsa-mir-26a-5p**	hsa-mir-125b-5p*	hsa-mir-205-5p	

Table S2. Primer sequences for qPCR.

Gene	Forward (5′→ 3′)	Reverse (5’ → 3’)
ACTB	TTCCTGGGCATGGAGTC	CAGGTCTTTGCGGATGTC
BCL2	GATAACGGAGGCTGGGATGC	GGCAGGCATGTTGACTTCAC
CDH1	CTCCTGAAAAGAGAGTGGAAG	GCAGTGTCTCTCCAAATCCG
ERBB2	GGCACAGTCTACAAGGGCAT	AGGGCATAAGCTGTGTCACC
HPRT1	TGACACTGGCAAAACAATGCA	GGTCCTTTTCACCAGCAAGCT
INPPL1	ATGCCTCAGATGGGGAGGAT	CTCACAGCTTCCAGGTCCAG
ITGA5	GCCGATTCACATCGCTCTC	TCTTCTCCACAGTCCAGCAAG
MMP13	CCTTGATGCCATTACCAGTCTCC	AAACAGCTCCGCATCAACCTGC
TBP	TTCGGAGAGTTCTGGGATTGTA	TGGACTGTTCTTCACTCTTGGC
VEGFA	CATGCCAAGTGGTCCCAGG	GGTCTCGATTGGATGGCAGT
ZEB1	ACCTGCCAACAGACCAGAC	TTCCTGTGTCATCCTCCCAG
ZEB2	CCACGATCCAGACCGCAAT	CCAGGTGGCAGGTCATTTTCT

Table S3. miR-205 target genes and function.

Target gene	Gene function	Association to cSCC carcinogenesis
BCL2	Membrane protein of the outer mitochondrial membrane	Inhibition of apoptosis [65]
ERBB2	Receptor tyrosine kinase	Cell growth, survival [66]
INPPL1	Phosphatase	Inhibition of cell migration in keratinocytes [42]
ITGA5	Integrin	Migration, survival [67]
MMP13	Proteinase	Proliferation, migration, invasion [68]
VEGFA	Growth factor	Proliferation, survival [65]
ZEB1	Zink finger protein	EMT induction [69]
ZEB2	Zink finger protein	EMT induction [16]
