# Supplementary figures and images for: Knockdown of Simulated-Solar-Radiation-Sensitive miR-205-5p Does Not Induce Progression of Cutaneous Squamous Cell Carcinoma In Vitro
Source: Int J Mol Sci. 2023 Nov 17;24(22):16428. doi: 10.3390/ijms242216428 (PMC10671527; doi:10.3390/ijms242216428)

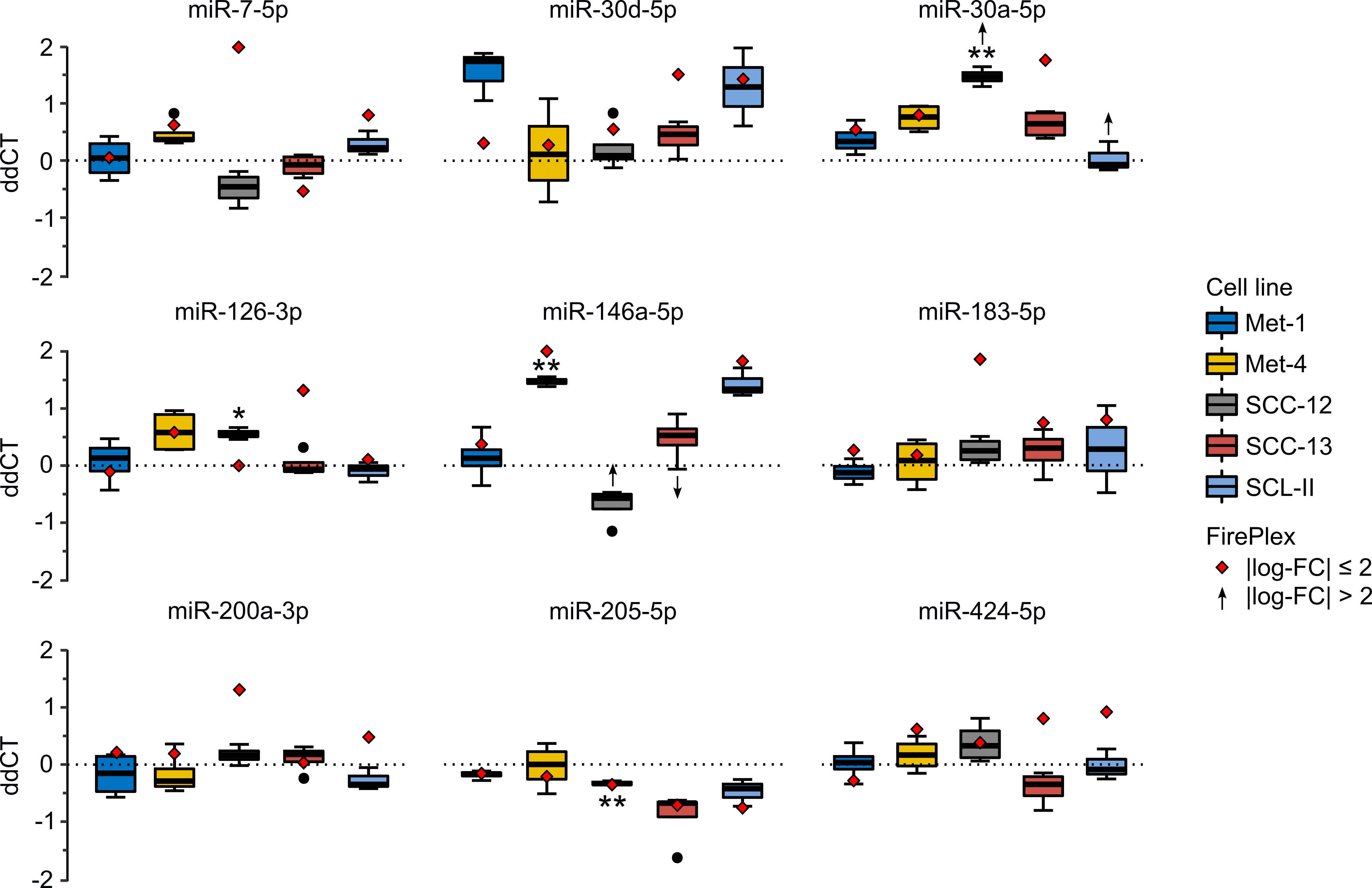

Supplement: Supplementary file 1 [file ijms-24-16428-s001.zip › FigureS1.png]

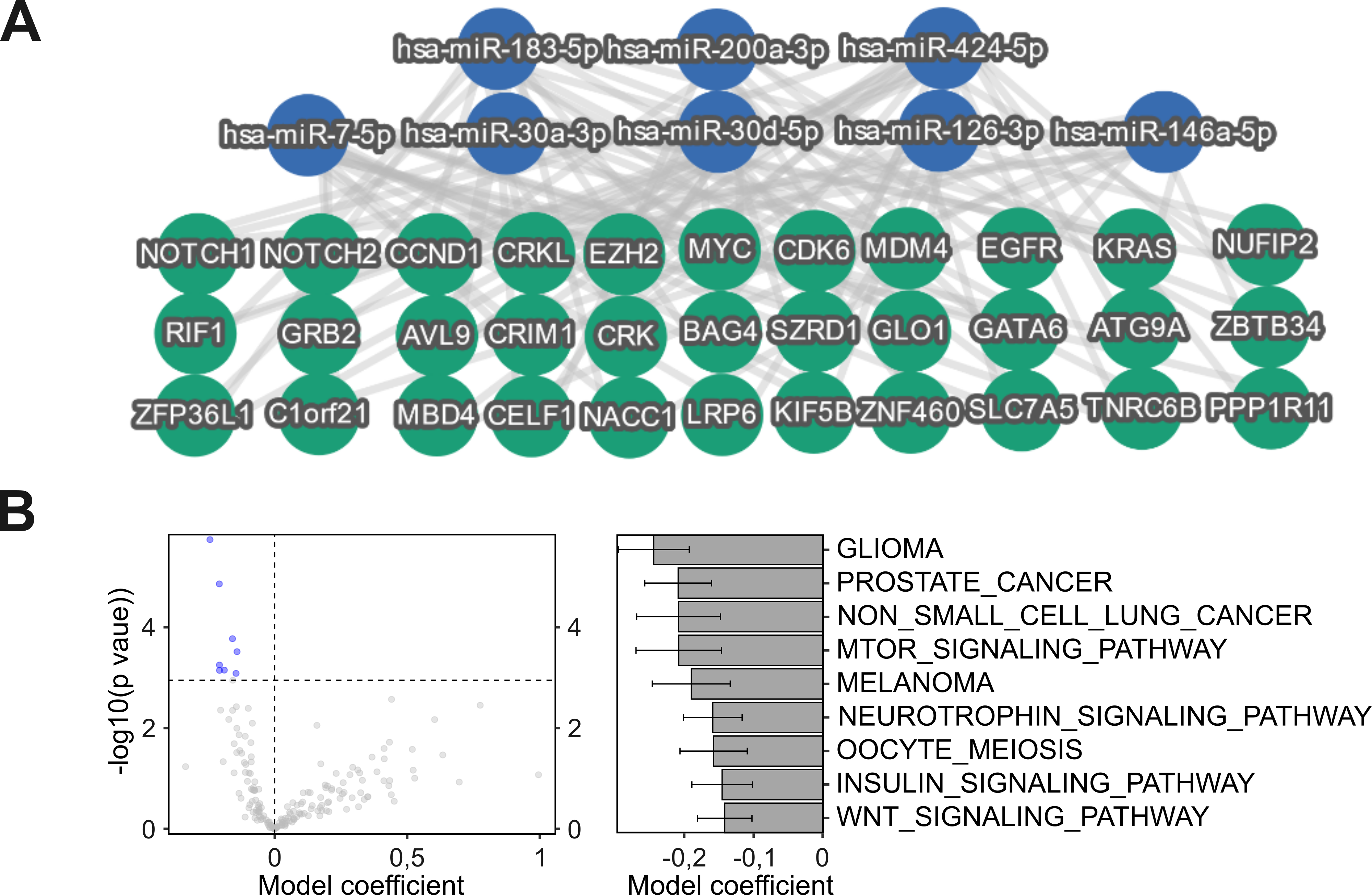

Supplement: Supplementary file 1 [file ijms-24-16428-s001.zip › FigureS2.png]
